# A Semisolid Polymer-Based Electrochemical Cell for Electrostimulated Lactate Detection

**DOI:** 10.1021/acsomega.5c09923

**Published:** 2025-12-11

**Authors:** Carolina Vitales, Jordi Sans, Adrián Fontana-Escartín, Elaine Armelin, Carlos Alemán

**Affiliations:** † IMEM-BRT Group, Departament d’Enginyeria Química, EEBE, 16767 Universitat Politècnica de Catalunya, C/Eduard Maristany 10-14, Building I, Second Floor, 08019 Barcelona, Spain; ‡ Barcelona Research Center for Multiscale Science and Engineering, Universitat Politècnica de Catalunya, C/Eduard Maristany 10-14, Building I, Basement, 08019 Barcelona, Spain; § Institute for Bioengineering of Catalonia (IBEC), The Barcelona Institute of Science and Technology, Baldiri Reixac 10-12, 08028 Barcelona, Spain

## Abstract

A fully organic electrochemical cell was designed for the immobilization of l-lactic acid (LLA) and for the detection of lactate (LA) oxidation to pyruvate, representing a nonenzymatic sensor for *in vitro* LA analyses. The novelty relies on the preparation of a bilayer semiconducting electrode, composed of an inert and biocompatible poly­(propylene) (PP) film and a coating of doped poly­(3,4-ethylenedioxythiophene) (PEDOT), which served as both working and counter electrodes, for the monitoring of the LA/pyruvate redox reaction and the quantification of these biomolecules using electrochemical tools and UV–visible spectroscopy, respectively. An advantageous characteristic of PP/PEDOT-LA electrodes was the faster pyruvate release under electrical stimulation (by pulsed short-time voltage applications), reaching 100% conversion in 360 min. Such results were promoted by the replacement of aqueous electrolytes for a highly stable hydrogel, constituted of poly­(γ-glutamic acid) (γ-PGA) cross-linked with cystamine, which was employed as a porous and permeable semisolid electrolyte in the polymer-based electrochemical cell (PEC). The resulting insights would help to improve the analytical methods currently employed for the quantification of LA in the biomedical, food, and beverage industries.

## Graphical abstract

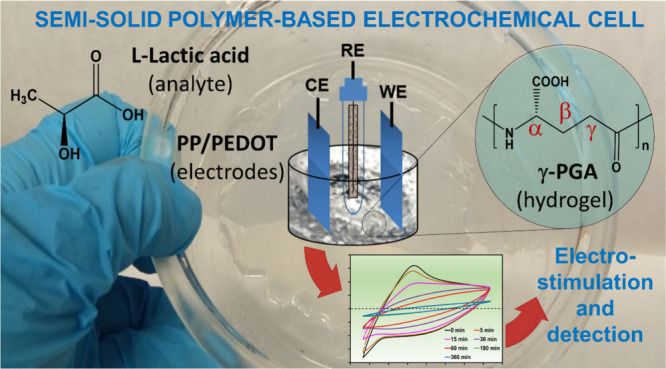

## 1 Introduction

Currently, there is an enormous effort by the scientific community to obtain fully organic electrochemical cells (OECs) as a sustainable alternative to fabricating supercapacitors, batteries, and sensing devices free of transition metals and other critical raw materials.^1,2^ This was mainly motivated by some concerns related to chemical safety aspects (toxicity, handling, recyclability, and metals recovery after end-life) and the negative social impact caused by the extraction of such feedstocks from vulnerable countries, with poor laws and human rights.^3,4^ A promising alternative is the employment of polymer-based redox materials, such as gel polymer electrolytes (GPEs),^5–7^ and polymer-based semiconducting electrodes made up of conducting polymers (CPs).^8,9^

Since most hydrogels and CPs, such as poly­(γ-glutamic acid) (γ-PGA) and poly­(3,4-ethylenedioxy­thiophene) (PEDOT), are highly biocompatible for manufacturing implantable sensors,^10^ they have also been employed in semisolid electrolyte compounds for biochemical applications. Nevertheless, polymer-based redox materials still face significant challenges, one of which is the combination of biocompatible electrodes and biodegradable electrolytes, with nontoxic byproducts and good electrochemical response for the target molecules desired to be quantified.^11–13^ For instance, Puiggalí-Jou et al. developed a fully biocompatible free-standing organic electrode employed as a platform for the controlled release of l-lactate (LA) under electrical stimulation.^14^ The combination of poly­(lactic acid) (PLA) layers with PEDOT allowed the controlled release of lactate molecules, induced by applying biphasic electrical pulses through the CP electrode. More recently, we have reported an optimized platform consisting of lactate-loaded PLA microfibers, intercalated with a PEDOT conducting thin film, for the sustained release of lactate for cardiac tissue regeneration.^15^ Other similar studies^16,17^ remark on the prompt of hydrogels and PEDOT to serve as good vehicles for LA controlled delivery.

This small biomolecule plays an important role in several intracellular reactions, such as the cellular respiration process, glucose production, inflammatory regulation, and molecular signaling.^18–20^ Thus, lactate is now considered to be an important regulatory molecule of intermediate metabolism involved in a variety of diseases, and their successful treatments are strictly related to the quantification of LA levels and its administration in clinical treatments.^21,22^ Currently, the lactate concentration in blood can be measured with point-of-care devices, based on oxidase enzymes,^23^ using incubation protocols and bioluminescence techniques, with an accuracy of very low concentrations (0.20 to 200 μM). Enzyme-based sensors, while highly effective due to their high selectivity and sensitivity, face several significant limitations regarding their being affixed to the electrode and their instability due to the enzyme’s sensitivity to pH or temperature shifts.^24,25^ Meanwhile, in clinical treatments, exogenous lactate administration follows oral and intravenous injections, which are limited either by the drug’s solubility in water or by the membrane permeability and biological stability.^26,27^

The challenge is how to design an electrochemical system, biocompatible and free of enzyme dependence, with good capacity of ionic and electrical conductivities to be used as an effective biosensor of biomolecule analytes or as a biomedical monitoring tool. Many times, technological advances require the combination of synergystic strategies. For instance, Mecerreyes and co-workers recently reported a 3D-printed PEDOT-based hydrogel as a long-term bioelectrode in electrocardiography (ECG) and electromyography (EMG) devices, exhibiting excellent health monitoring over 2 weeks, after muscle electrostimulation.^28^ In another example, Pérez-Madrigal et al. investigated the use of γ-PGA, a biocompatible, edible biopolymer and nonimmunogenic and biodegradable material, as a polyelectrolyte hydrogel for organic electrochemical supercapacitor (OESC) applications.^29^ The hydrogel was assembled between two layers of PEDOT CP, electrogenerated by chronoamperometry, obtaining good and stable storage charges and charge–discharge capacitances. Mustafa and Leese developed an enzymatic-free molecularly imprinted polymer (MIP) sensor by capturing lactate molecules, with proven sensitivity toward the presence of similar acidic organic molecules.^30^ The challenge of nonenzymatic LA biosensor advancement also finds utility in the food industry, for example, in monitoring fermentation processes in dairy products and in preventing spoiled food stocks from reaching consumers.^31,32^ Inspired by such previous studies, in this work, we have adapted the reported approaches for developing a new electrochemical device for the analytical detection of LA.

Herein we report the preparation of a semisolid polymer-based electrochemical cell (hereafter denoted ss-PEC) composed of a γ-PGA porous hydrogel network, as a polyelectrolyte, and of two electrodes, which are totally organic-based and free of metals, representing the anode and the cathode. They were strategically designed to sustain an electrochemically generated PEDOT/4-dodecylbenzenesulfonic acid (DBSA) layer on a polypropylene (PP) substrate by cold-plasma activation technology.^33,34^ Oxygen plasma activation is a highly effective surface modification technique for polymers, offering numerous advantages, particularly in enhancing the adhesion and wettability. In our previous studies, we demonstrated that well-controlled parameters of the plasma process allow the creation of energetic species in nonpolar bonds (like C–H or C–C) of the PP polymer and replace some of them by grafting oxygen-containing functional groups such as radical species (−O*), carbonyl (C=O), and carboxyl (−COOH) bonds, without damage to the polymer matrix bulk properties.^35–39^ The enzyme-free system was designed to act as both a carrier of l-lactate molecules and an electrochemical sensor for the quantification and monitoring of l-lactate delivery through the gel, under electrostimulation. Thus, in this first approach, the feasibility of the cell will be demonstrated. However, important aspects such as the sensitivity and selectivity of the system toward other biomolecules that can actuate as interferents to LA oxidation to pyruvate as well as the limits of LA concentration detection (LOD) shall be approached in future investigations as a proof of concept of the nonenzymatic PEDOT-based sensor performance.

## 2 Materials and Methods

### 2.1 Materials

Isotactic polypropylene pellets (PP) (*M*_n_ = 50 000 g/mol, *M*_w_ = 190 000 g/mol, and polydispersity index = 3.80), 3,4-ethylenedioxythiophene (EDOT; 97%), lithium perchlorate (LiClO_4_), acetonitrile (99.8%), ammonium persulfate (APS; 98%), 4-dodecylbenzenesulfonic acid (DBSA, 70 wt % in isopropanol), cystamine dihydrochloride (Cys; ≥98.0%), 1-[3­(dimethylamino) propyl]-3-ethylcarbodiimide methiodide (EDC), and phosphate-buffered saline (PBS) solution were purchased from Sigma-Aldrich (USA). LiClO_4_ was stored in an oven at 80 °C before its use in anodic polymerization. l-(+)-Lactic acid (LLA) (IUPAC: 2-hydroxypropanoic acid) was purchased from Honeywell Fluka (Fisher Scientific, Sweden), and sodium bicarbonate (NaHCO_3_) was purchased from Panreac Quimica S.A.U. (Barcelona, Spain). Free-acid poly-γ-glutamic acid (γ-PGA), with an average molecular weight of 350 000 g/mol, was acquired from Wako Chemicals GmbH (Neuss, Germany). An analytical test kit for the detection of l-lactate by the UV method (AK00131) was acquired from NZYTech, Ltd.

### 2.2 Preparation of Polypropylene Films and Surface Activation with Cold Plasma

The multifunctional ss-PEC system, employed as an electrostimulated carrier of LA molecules, was prepared following the steps represented in Scheme 1. First, PP films were obtained by pressing the pellets in a hydraulic press with a 15-ton capacity and Atlas series heated platelets (Scheme 1A, step 1). For this purpose, 3.7 g of PP pellets was weighed and placed in a square mold of 6 × 6 cm^2^, made with Teflon and aluminum foil, and moved to the press jaws. Afterward, the material was held at 180 °C for 5 min, and increasing pressure (from 0 to 5 tons) was applied and maintained for 2.5 min. Then, the pressure was augmented to 7 tons and preserved for another 2.5 min. Finally, the film was removed from the press jaws and cooled to room temperature before use.

**Scheme 1:**
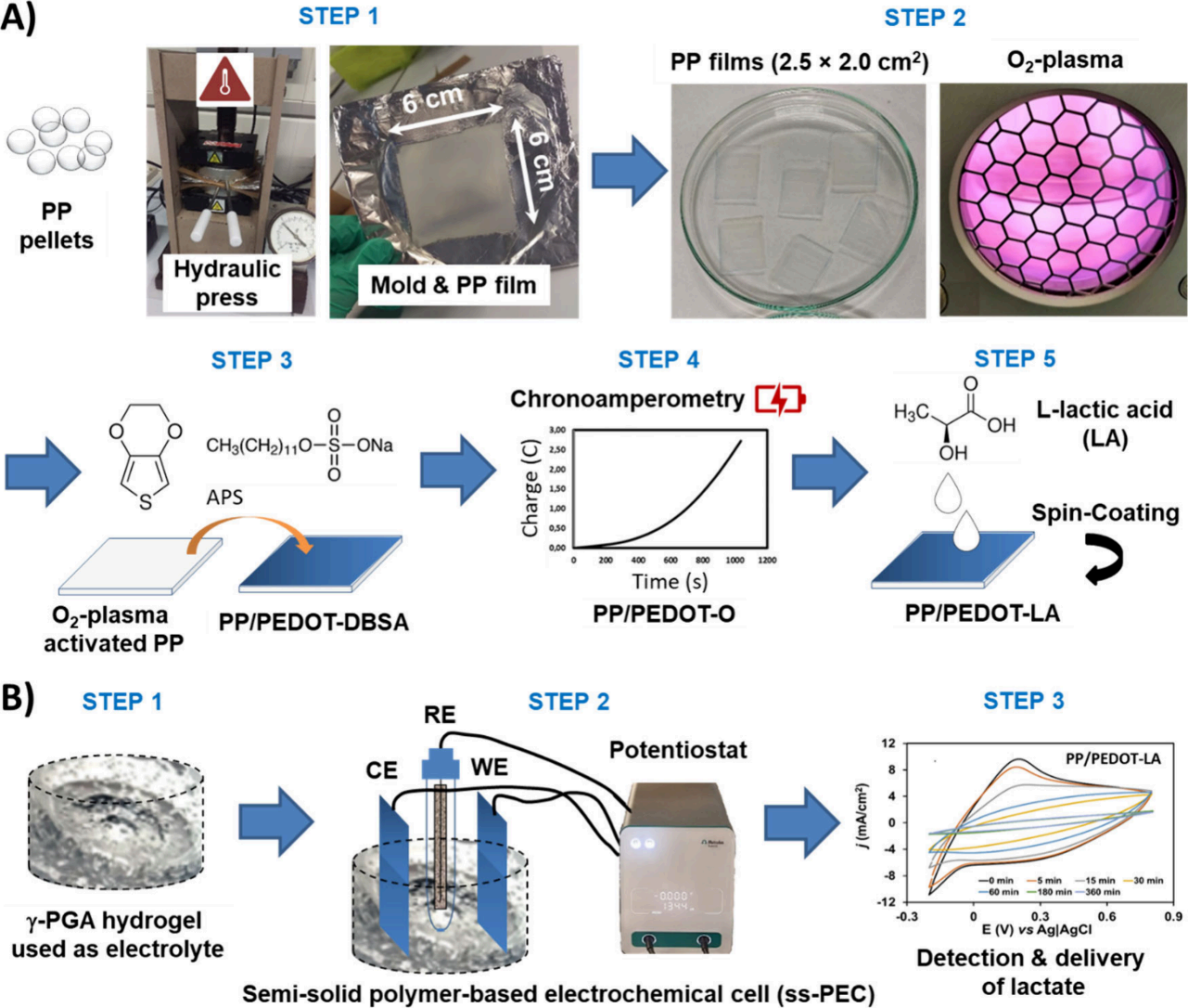
Construction of the Fully Organic Electrochemical Cell (ss-PEC); (A) Scheme of the Different Steps Used to Prepare the Semiconducting Electrodes: (1) Composed of a Substrate of PP Film, Manufactured by Thermal Pressing; (2) Activation of the Substrate with Plasma; (3) Coating with PEDOT CP Obtained by Chemical Oxidation; (4) PEDOT Electrochemically Oxidized with LiClO_4_; and (5) Loading with LA Molecules; (B) Assembly of the ss-PEC: (1) Preparation of the γ-PGA Hydrogel as a Polyelectrolyte; (2) Mounting of the Semiconducting Electrodes in the Polyelectrolyte Medium; and (3) Utilization of the ss-PEC in the Detection and Electrostimulation Delivery of LA

The resulting PP films were cut into 2.5 × 2.0 cm^2^ dimensions and washed in 30% ethanol aqueous solution before the surface activation with O_2_-plasma (Scheme 1A, step 2). The rectangular films were moved to a chamber of 25 dm^3^ capacity in an LFG generator (Diener Electronic GmbH Co., Germany), equipped with low-pressure radio frequency (RF) plasma (80 MHz) and with a maximum power supply of 1000 W. The system was purged up to 0.18 mbar of vacuum pressure and subsequently filled with oxygen gas with a pressure of 0.30 mbar using a gas flow fixed for 180 s, following our previously optimized procedure.^35^ The power discharge was 250 W. The PP plasma-activated surfaces were used as substrates for the chemical polymerization of the PEDOT conducting polymer.

### 2.3 Preparation of PP/PEDOT Semiconducting Electrodes

PEDOT doped with DBSA was deposited on the activated PP films by direct oxidative chemical polymerization (Scheme 1A, step 3). For this purpose, PP samples were immersed in a micellar solution containing 127 μL of DBSA and 24.7 mL of Milli-Q water. This solution was stirred for 1 h at 500 rpm and 40 °C before the addition of 149 μL of EDOT monomer. It was left 1 h further for mixing, under the same conditions, followed by the slow addition of 0.36 g of the initiator promoter (APS), previously dissolved in 2 mL of Milli-Q water. The reaction temperature and stirring conditions were maintained at 40 °C and 500 rpm, respectively. After 24 h of chemical reaction, the formation of PEDOT over PP film was evidenced by the color change to dark blue, as reported in our previous work.^40^ The resulting semiconducting electrode, named PP/PEDOT-DBSA, was removed from the reactor vessel, washed three times with Milli-Q water and acetone to eliminate any unreacted monomer and oxidant molecules, and dried under vacuum for 24 h while being protected from light.

An additional PEDOT conducting pathway was achieved by applying chronoamperometry to the EDOT solution with LiClO_4_ as the oxidant agent. For this purpose (Scheme 1A, step 4), PP/PEDOT-DBSA films acted as working electrodes (WEs) during electrochemical anionic polymerization. The reaction was carried out in a three-electrode cell filled with 10 mL of an acetonitrile solution containing 0.05 M EDOT monomer and 0.1 M LiClO_4_ as the supporting electrolyte, applying a constant potential of +1.40 V and adjusting the polymerization charge to 2.7 coulombs. The electrochemical polymerization was performed using a VersaStat II potentiostat–galvanostat connected to a computer controlled through a Power Suite Princenton Applied Research program, with a reference electrode (RE) of Ag|AgCl containing a KCl saturated aqueous solution (*E*° = 0.222 V at 25 °C) and Pt wire as the counter electrode (CE). The semiconducting organic electrodes were identified as PP/PEDOT-O, remembering that “O” refers to the outer layer, which is composed of a PEDOT/LiClO_4_-doped system. The area of the WE was delimited to 0.25 cm^2^, and the current is expressed as the current density (mA/cm^2^).

### 2.4 Adsorption of l-Lactic Acid to the Semiconducting Organic Electrodes

PP/PEDOT-O films were employed as all organic electrodes (i.e., as WE and CE) for the electrochemical detection of lactate (LA) anions and as an electro-stimulated electrode for LA delivery. Therefore, LLA was loaded onto the PP/PEDOT-O surface by applying a spin coating to a previously prepared LLA aqueous solution (1.5 M). A rotational speed of 900 rpm was applied for 60 s (Scheme 1A, step 5). For this step, a WS-400BZ-6NPP spin-coater (Laurell Technologies Co.) was employed. The acquired samples were labeled as PP/PEDOT-LA. The presence of LA molecules on electrode surfaces was analyzed by Raman single point and mapping (section 2.6).

### 2.5 Assembly of the Semisolid Polymer-Based Electrochemical Cell (ss-PEC)

The construction of the fully organic electrochemical cell (ss-PEC) was performed using the γ-PGA porous hydrogel as a semisolid electrolyte (Scheme 1B, step 1), which was prepared following the method previously reported by our group.^29,40,41^ The chemical synthesis is summarized in Scheme S1. In brief, γ-PGA was dissolved in 1 mL of 0.5 M NaHCO_3_ at 4 °C and 500 rpm for 10 min. Then, 1-[3­(dimethylamino)­propyl]-3-ethylcarbodiimide methiodide (EDC) was added to the solution and mixed under the same conditions for 2 min. Afterward, the process was repeated adding cystamine dihydrochloride (Cys) for covalent cross-linking. The final solution, with a molar ratio of 5:4:2 γ-PGA:EDC:Cys, was left to gel, at room temperature for 20 min, into circular molds with a diameter and height of 1 cm. Two PP/PEDOT-O electrodes, placed parallel in the γ-PGA solution before its gelation, were used for assembling the ss-PEC (Scheme 1B, step 2). LA delivery experiments were executed using PP/PEDOT-LA as WE and PP/PEDOT-O as CE. Finally, the electrochemical experiments for the electrostimulation of LA delivery were performed (Scheme 1B, step 3), employing the potentiostat–galvanostat equipment described for CA assays.

### 2.6 Physical–Chemical Characterization

The systems derived from each step were characterized using chemical, physical, and morphological characterization techniques, and the functionality of the semiconducting electrodes and the LA release from the ss-PEC device were analyzed by electrochemical methods.

Fourier transform infrared (FTIR) was used to detect the main organic functional groups in polar polymers. The spectra were recorded on an FTIR Jasco 4700 spectrophotometer equipped with an attenuated total reflection accessory (top plate) with a diamond crystal (Specac model MKII Golden Gate Heated Single Reflection Diamond ATR). The samples were evaluated using Spectra Manager software, and for each sample, 32 scans were performed between 4000 and 600 cm^–1^ with a resolution of 4 cm^–1^.

Raman spectroscopy was employed to determine the chemical composition of the polymers used in the present study. For that, a commercial Renishaw inVia Qontor confocal Raman microscope was chosen, with laser sources of 532 nm for thermoplastic characterization and 785 nm for fluorescence interference from conducting polymer-based samples. The equipment is connected to a Leica DM2700 M microscope and a CCD detector (1200 lines·mm^–1^ grating). The exposure time was 10 s, and the laser power was adjusted to between 0.5 and 10% of its nominal output power. General spectra were collected in the extended range of 600–3600 cm^–1^ with 5–10 accumulations. To confirm the proper loading of LA molecules to the organic PP/PEDOT electrode, high-resolution spectra in the 2550–3100 cm^–1^ region were acquired by reducing the exposure time to 1 s and increasing the nominal output power and total number of accumulations to 50% and 200, respectively. Finally, in some steps of electrode preparations, Raman maps of 85 points × 55 points (total of 4620 acquisitions) were collected to ensure the multilayer electrode construction regarding the homogeneous loading of lactate molecules (optical images).

The roughness of the organic electrodes was obtained by using a Dektak 150 stylus profilometer (Veeco, Plainview, NY). Imaging of the films was conducted using the following optimized settings: tip radius = 2.5 μm, stylus force = 3.0 mg, and length = 1.5 μm for 30 s (i.e., speed of 0.05 μm/s).

The morphology of the samples was observed by scanning electron microscopy (SEM), employing a focused ion beam Zeiss Neon40 scanning electron microscope operating at 3 or 5 kV. All samples were sputter-coated with a thin carbon layer using a K950X turbo evaporator to prevent electron charging problems. The averages of particle and pore sizes were calculated from at least 100 measurements (*n* = 100) in different images obtained by SEM.

### 2.7 Electrochemical Assays and LA Delivery Detection

#### 2.7.1 Cyclic Voltammetry

The electrochemical performance of the organic semiconducting electrodes was studied by cyclic voltammetry (CV), using an Autolab PGSTAT302N potentiostat–galvanostat and NOVA 2.1 software. The experiments were conducted with two different setups: (i) in a three-electrode cell filled with a 0.1 M phosphate buffer saline (PBS) solution (pH 7.4), as the liquid electrolytic solution and using Ag|AgCl (KCl, 3 M) as the reference electrode (RE) and (ii) in a two-electrode cell with γ-PGA, cross-linked with cystamine and having hydrogen carbonate as counterions, as a soft and porous polyelectrolyte. In both cases, the CE was constituted of PP/PEDOT-O films and the WE was the LA-loaded counterpart (PP/PEDOT-LA). The two-electrode cell with the semiconducting organic electrodes faced and the γ-PGA polyelectrolyte forms the ss-PEC system developed in the present study. In all cases, 25 consecutive oxidation–reduction cycles were recorded at a scan rate of 50 mV/s with an initial/final potential of −0.2 V and a reversal potential of +0.8 V to avoid water oxidation reactions. CVs at different scan rates (10, 25, 100, and 200 mV/s) were performed to access the scan-rate dependence of the LA oxidation peak.

The ability to exchange charge reversibly (i.e., electrochemical activity or electroactivity, EA) and the electrochemical stability were determined through direct measurement of the anodic and cathodic areas between cyclic voltammograms from the 2nd and 25th cycles. The loss of electroactivity (LEA, in%) is expressed by eq 1

1 $$LEA\left(\%\right)=\frac{\Delta Q}{Q_{2}}\times 100$$

where Δ*Q* is the difference in the voltammetric charges (in C) between the second redox cycle (*Q*_2_) and the last cycle (*Q*_25_).

#### 2.7.2 Chronoamperometry

The LA release from the PP/PEDOT-LA electrodes in an ss-PEC was electrostimulated by chronoamperometry (CA) and quantified by UV–vis spectroscopy. The electrostimulation applied was made through biphasic pulses of positive and negative (±) voltages of 0.2 V, with a duration of 5 ms each and a total time of 15 min. Afterward, CV was carried out for each time interval (5, 15, 30, 60, 180, and 360 min).

The concentration of lactate released from the ss-PEC was quantified using an indirect method (i.e., it was promoted by an analytical test kit based on the spectrophotometric measurement of nicotinamide-adenine dinucleotide (NADH) formed through the combined action of l-lactate dehydrogenase (l-LDH)). The concentration of NADH was measured at 340 nm with a UV–vis Cary 100 Bio spectrophotometer (Agilent, Santa Clara, CA, USA). All electrochemical assays and LA release experiments were replicated at least three times (*n* = 3).

## 3 Results and Discussion

### 3.1 Chemical Structure and Morphological Description of the Organic Conducting Electrode and the Polyelectrolyte Employed in the Construction of the ss-PEC

The engineered semiconducting electrodes (PP/PEDOT-O and PP/PEDOT-LA) were constituted by a nonbiodegradable but biocompatible polymer, and the soft and porous electrolyte was made with a biocompatible, biodegradable, and nontoxic hydrogel (γ-PGA). Thus, the design of the ss-PEC, intended to be used as a vehicle for the electrostimulated delivery of lactate, is a fully biocompatible system. The main chemical structures of the organic molecules used to design the ss-PEC are presented in Figure 1A.

**Figure 1:**
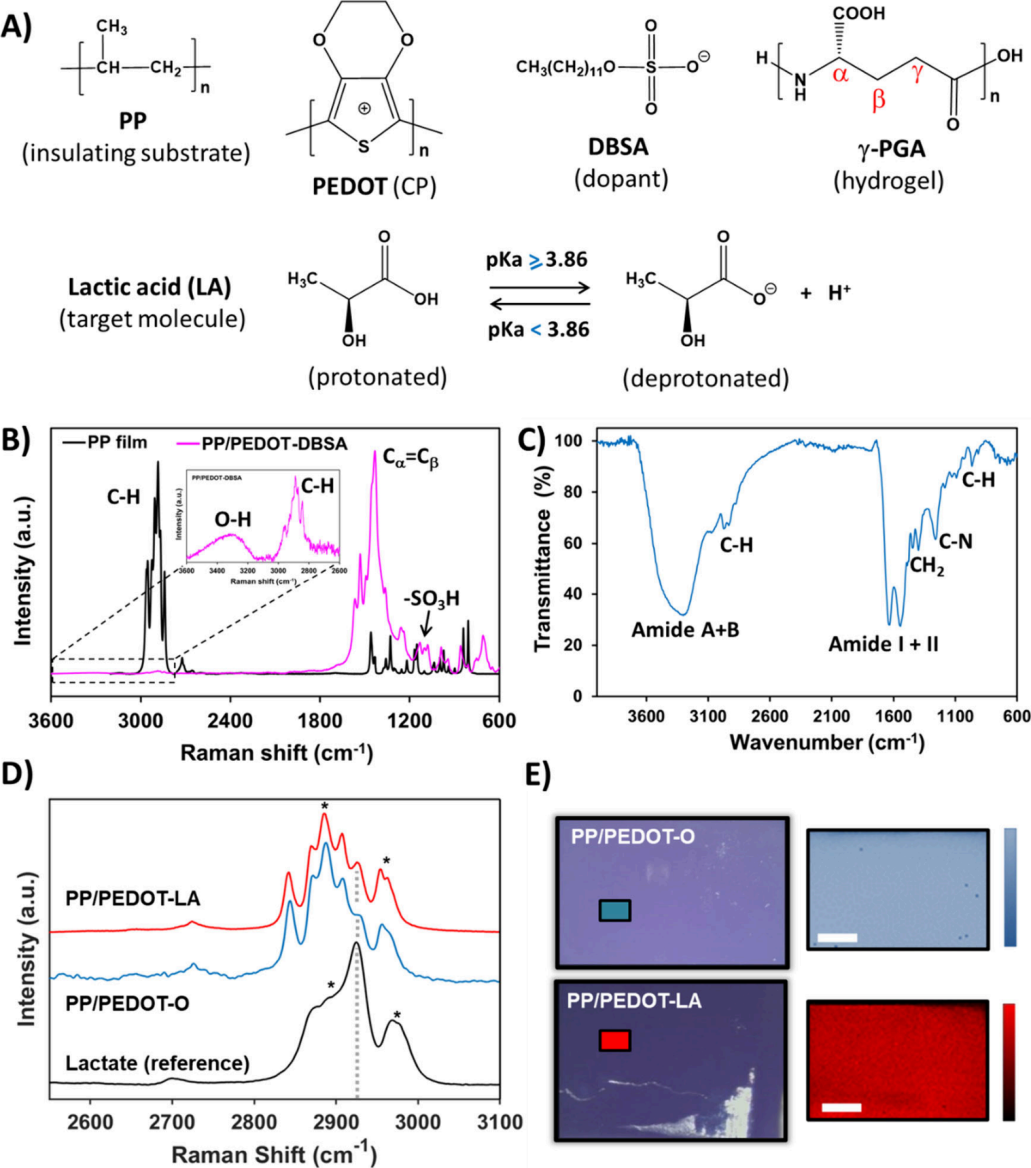
A) Chemical structures of all organic compounds employed to prepare the ss-PEC. B) Raman spectra of the PP film and functionalized PP/PEDOT-DBSA. The inset spectrum highlights the 3600–2600 cm^–1^ region of the PP/PEDOT-DBSA sample, which was screened with reduced exposition time to avoid CP interference. C) FTIR spectrum of the γ-PGA hydrogel used as a semisolid electrolyte in ss-PEC. D) High-resolution Raman spectra of the 2550–3100 cm^–1^ region carried out for PP/PEDOT-LA, PP/PEDOT-O, and LA (reference) samples. Asterisks indicate the overlapped absorption bands. E) Raman maps acquired for PP/PEDOT-LA and PP/PEDOT-O samples (on the left) and detail of the inset zones (on the right). Scale bar of the optical images (white bar): 66 μm.

For electrode preparation, it was important to ensure the deposition of a semiconducting layer of CP for the electrochemical stimulation tasks. The presence of counterions, such as DBSA, in intrinsically semiconducting polymers is of fundamental relevance to ensure the creation of polarons and bipolarons in the conjugated polymer chains. Therefore, Raman spectroscopy was chosen as the best technique to discriminate the nonpolar (C=C) and low polar (C–S–C) linkages of CP from the low polar groups (C–H) of the polymer insulating matrix (PP). The Raman spectra of the PP film and PP/PEDOT-DBSA are shown in Figure 1B. The most relevant bands of the PP film are identified as stretching vibrations from the symmetric and asymmetric stretching of C–H (in −CH_2_– and −CH_3_ groups) at around 2800–2900 cm^–1^. The peak at 1457 cm^–1^ is attributed to −CH_2_– bending vibrations, whereas rocking is observed at ∼800 cm^–1^. The absorption bands positioned at 1000 and 842 cm^–1^ are specific from isotactic PP chains and usually do not appear in the syndiotactic or atactic polymer. A peak at around 1356 cm^–1^ (medium intensity) corresponds to the deformation (or wagging) of the −CH_3_ groups. Therefore, the plasma treatment was proven not to damage the PP surface, as the plasma conditions are well controlled, corroborating our previous studies with this polymer.^42^

On the other hand, the presence of PEDOT dominates the Raman spectrum in Figure 1B, when it is immediately deposited on the PP oxygen-plasma-activated surface. As explained in our previous work,^43^ this effect occurs when the incident radiation coincides with the frequency of an electronic transition of the sample. The broad and intense absorption band at 1432 cm^–1^ is associated with the C_α_=C_β_ symmetric stretching vibrations, the sharp peak at 1530 cm^–1^ is assigned to asymmetric stretching of C_α_=C_β_, and the peaks at 1364 and 1253 cm^–1^ are attributed to the intra-ring C_β_–C_β_ and inter-ring C_α_–C_α_ stretching vibrations of the CP, respectively. The band at 988 cm^–1^ is related to oxyethylene ring deformation, and the peaks at 860 and 695 cm^–1^ are associated with the asymmetric and symmetric C–S–C deformations, respectively.^44–46^ On the contrary, polar groups, like the one belonging to the dopant molecule DBSA (−SO_3_H), are more difficult to identify by Raman, with only a small shoulder corresponding to O–H linkages being visible in the inset of Figure 1B.

For polar polymers, infrared spectroscopy is preferred with respect to Raman spectroscopy since the absorption bands of polar functional groups are accentuated. Figure 1C shows the typical absorption bands of the cross-linked γ-PGA hydrogel. The broad and strong absorption band appearing at 3650–3000 cm^–1^ is clearly identified from the hydrogen bonding interactions among the C=O and N–H groups (amide A and B stretchings). The success of the polymerization cross-linking is evidenced by the disappearance of the free carboxylic acid (from the raw material) that usually appears at 1730 cm^–1^, as previously reported.^41,47^ The sharp and intense doublet peaks of amide I (1633 cm^–1^) and amide II (1544 cm^–1^) are also correlated with the cross-linking of γ-PGA chains, promoted by cystamine bridges (Scheme S1). On the other hand, bonds involving sulfur (C–S and S–S) could not be detected by FTIR and were identified by Raman (Figure S1).^48^

The successful loading of LA was confirmed by acquiring single-point high-resolution Raman spectra of PP/PEDOT-LA, as compared with PP/PEDOT-O and LA (reference) samples (Figure 1D). As can be observed, the main characteristic peak of LA at 2926 cm^–1^ (C–H stretching vibration) can be clearly identified for the PP/PEDOT-LA sample. Furthermore, the effect of the wide shoulder and secondary vibrational modes of l-lactate at about 2895 and 2968 cm^–1^, respectively, can be also observed in the PP/PEDOT-LA spectrum (marked with an asterisk in Figure 1D). Such features are attributed to the contribution of the symmetric and asymmetric νCH_3_ and ν­(C–H) vibrational modes. As can be seen, the bands overlap with other C–H groups from the substrate composition. Finally, the homogeneity of the LA loading is demonstrated through the analysis of the Raman maps (Figure 1E). To do so, the multiple-point spectral acquisitions have been compared with the high-resolution measurements performed in Figure 1D. Accordingly, a brighter color bar indicates higher spectral similarity and thus confirmation of the proper multilayer construction.

Topography and sample roughness may play an important role in the performance of the ss-PEC since a heterogeneous distribution of PEDOT CP may hinder LA delivery by electrical stimulation. On the other hand, several studies proved that the morphology of doped-PEDOT usually favors the interaction of the semiconducting polymer with biomolecules.^49,50^ Therefore, complementary information about sample morphology was obtained by SEM at different magnifications (Figure 2). PP films exhibited a flat, compact, and smooth morphology (Figure 2A–C), which, after oxygen plasma treatment, was transformed into more roughness patterning (Figure S2A,B). Moreover, the process only created some radicals and polar sites in the PP molecular chains at the nanometric level and does not damage the molecular core structure of the PP plastic (Figure S2C), which was already observed in previous work and corroborated by other researchers with other thermoplastics.^42,51,52^ Consequently, the cold-plasma treatment uniquely affected the surface roughness (*R*_q_), changing from 1.4 ± 0.2 to 2.7 ± 0.5 μm for the pristine film and O_2_-plasma PP-treated samples, respectively. This step (Scheme 1A, step 3) was necessary to create functional sites for the adhesion of one layer of PEDOT-DBSA on the PP substrate (Figure 2D–F) and to ensure the obtaining of the fully organic semiconducting electrode after the electropolymerization by chronoamperometry (Scheme 1A, step 4). This strategy has been proven to be highly efficient to ensure the proper adhesion of the conducting polymer to different materials used as biomedical implants.^53–55^ As can be seen in Figure 2G–I, the morphology of the CP doped with LiClO_4_ was completely irregular, with a denser and flatter structure compared to that of PEDOT-DBSA. However, closer inspection of some zones revealed the presence of PEDOT-O in the format of nanoparticles integrated into the PP film and into the PEDOT-DBSA layer, visible in higher-magnification images (Figure 2I). Once the LA was spin-coated onto this rough and porous architecture, the biomolecules were completely absorbed, and the morphology of the PEDOT layer prevailed in the micrographs (Figure 2J–L).

**Figure 2:**
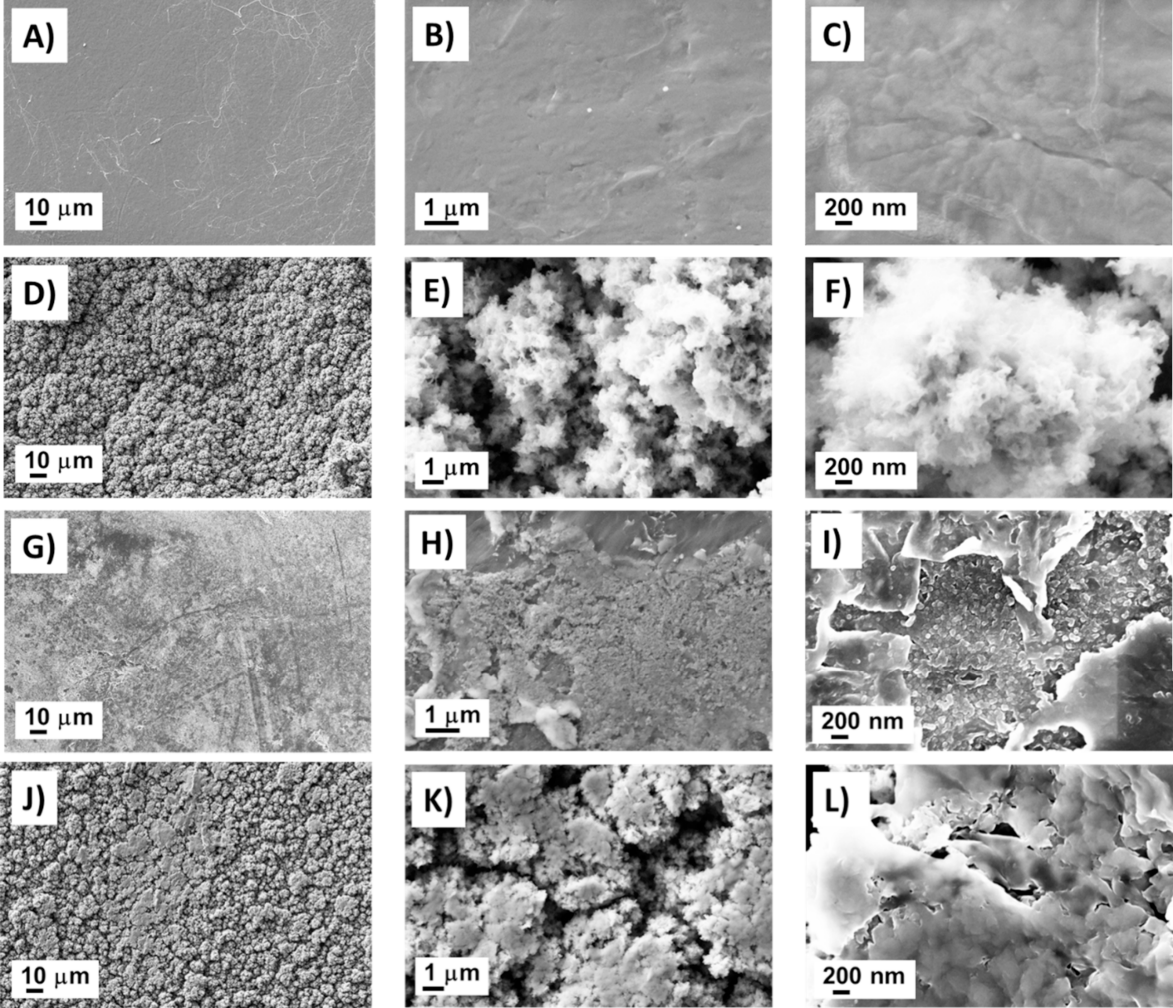
SEM micrographs of the A–C) PP flat film, D–F) PP/PEDOT-DBSA film, G–I) PP/PEDOT-O film, and J–L) PP/PEDOT-LA film surfaces. Scale bars represent, from left to right, low and high magnifications (500×, 10000×, and 25000×, respectively).

Finally, SEM images of the γ-PGA hydrogel showed a 3D network with interconnected regular pores with dimensions of 11.0 ± 3.0 μm and robust walls (Figure S3), supporting the fact that this hydrogel is an excellent candidate for good mass transport and diffusion phenomena in applications as semisolid electrolytes.^56–59^

### 3.2 Electrochemical Activity and Stability of the Organic Electrodes in ss-PEC

The functionality of PP/PEDOT-O as an organic semiconducting electrode and as a LA carrier was evaluated on the basis of its electroactivity (EA) (i.e., its ability to exchange charge with the electrolyte) and on the basis of its loss of electrochemical stability (LEA). Figure 3 presents the respective CV curves measured at a scan rate of 50 mV/s. As explained in section 2.4, two media were employed: physiological medium PBS (pH 7.4–7.6), which served as the liquid electrolyte in a three-electrode electrochemical cell;, and γ-PGA porous hydrogel (pH 4.5–5.0), which served as a semisolid electrolyte in ss-PEC (Scheme 1B). Although the cyclic voltammograms were slightly different, with the first being more capacitive, the current density (*j*) values and the electroactivity (EA) of the PP/PEDOT-O electrode, either in PBS or in γ-PGA electrolytes, were very similar (Figure 3A,B), reflecting a low electrical resistance between the electrode and the electrolytic medium.^58,60,61^ Besides, the loss of electroactivity (LEA), which was evaluated by summing the anodic and cathodic areas of the voltammograms from the 2nd and 25th consecutive redox cycles (eq 1), was also very similar in both media, upholding very low values (i.e., 3.4 ± 0.8 and 3.7 ± 1.8% in PBS and in γ-PGA hydrogel, respectively). Hence, the combination of reproducible electrochemical activity and the high electrochemical stability (low LEA) proved that both organic electrodes, composed of the PP substrate modified by the layer-by-layer deposition of PEDOT active compounds in ss-PEC, are very good candidates to replace metallic or ceramic electrodes.

**Figure 3:**
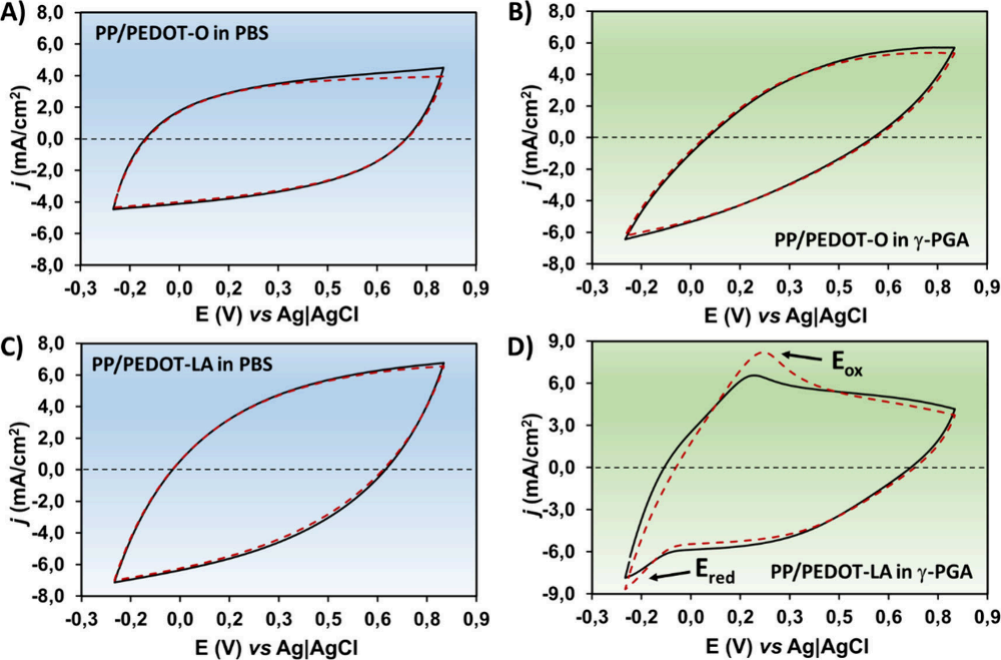
Second and 25th cycles of CVs with the A) PP/PEDOT-O working electrode (WE) in PBS (pH 7.4), as a conventional electrolyte in the three-electrode electrochemical cell, and B) PP/PEDOT-O WE in the γ-PGA hydrogel, as a novel semisolid electrolyte in ss-PEC. C) PP/PEDOT-LA in liquid PBS (pH 7.4) and D) PP/PEDOT-LA in the γ-PGA hydrogel were used as semisolid electrolytes to evaluate the electrochemical stability. The second CV voltammograms are represented with a continuous line, whereas 25 consecutive oxidation–reduction cycles are displayed by dotted lines. Scan rate: 50 mV/s.

The substitution of rigid materials by organic compounds is very attractive in the field of renewable energies,^62^ such as in the development of solid organic batteries and fuel cells, and in medicine, such as in bioimpedance sensor electronics. Regarding the first field mentioned, organic conducting electrodes can overcome the failure associated with the corrosion (microbiological corrosion, galvanic corrosion, localized corrosion, etc.) of active metals and the consequent decrease in the electrochemical cell efficiencies.^8^ In biosensors, the most important advantages rely on the low impedance characteristics and on the wearable adaptation to skin or to different organs of the human body.^63,64^ Moreover, the biocompatibility of such compounds was already demonstrated in numerous studies, such as the one mentioned in the Introduction.^65^ On the contrary, the most relevant disadvantage of organic electrodes is their poor mechanical integrity compared to that of solid metals and ceramic compounds and hence their short life span (i.e., they are most useful for single use or disposable electrodes).

Once the semiconducting electrode was proven to be stable and durable for repetitive measurements, the same experiments were carried out with the new organic electrodes charged with LA molecules. In general, the voltammograms of PP/PEDOT-LA, assayed in a conventional three-electrode cell and in PBS solution were similar in shape and current values (Figure 3C) to those of PP/PEDOT-O (Figure 3A), with the LEA being inappreciable. This proved the high reversibility of the electrode materials. Interestingly, faradaic behavior was observed when PP/PEDOT-LA electrodes were subjected to CV in γ-PGA electrolyte, revealing an oxidation peak at around +0.20 V, which was accentuated after 25 cycles (*E*_ox_ in Figure 3D). This peak is attributed to lactate oxidation to pyruvate (eq 2), which involves the conversion of the secondary alcohol to a ketone. Conversely, pyruvate is reduced to lactate by receiving two electrons and a proton at its central carbon (eq 3), which is supposed to remain trapped on the PP/PEDOT-O coating. The beginning of a reduction peak was also appreciated at around −0.20 V in the return curve (*E*_red_ in Figure 3D), corroborating the hypothesis that LA was converted to lactate by means of a pyruvate intermediate.^66,67^ This also confirmed the successful loading of LA in PP/PEDOT-O semiconducting electrodes, which received the electrons released by the redox reactions, acting as an electron carrier.

2 $$\left[\mathrm{oxidation}\right]L\text{‐}\mathrm{lactic acid}\left(\mathrm{LLA}\right)\to \mathrm{pyruvate}+2H^{+}+2e^{-}$$

3 $$\left[\mathrm{reduction}\right]\mathrm{pyruvate}+2H^{+}+2e^{-}\to \mathrm{lactate}\left(\mathrm{LA}\right)+H^{+}$$

The superior performance of γ-PGA, compared to that of a pure aqueous environment, as a host electrolyte for lactate conversion can be ascribed to its acidic pH nature (pH 4.5–5.0). At low pH, the reaction typically favors the conversion of lactate formation from pyruvate, as the accumulation of H^+^ can drive the equilibrium toward the production of lactate (which consumes H^+^), as can be seen in eqs 2 and 3. This is consistent with what happens under anaerobic conditions, in which lactate is produced to regenerate NAD^+^ and prevent acidosis, in cellular metabolism, catalyzed by the enzyme lactate dehydrogenase (LDH).

Therefore, one of the advantages of replacing physiological fluids (in our case, a buffer with controlled pH) with a semisolid polyelectrolyte with lower pH is the promotion of LA delivery and stabilization over time. In the biomedical field, it represents a challenge for the administration of LA in the stomach, where the pH reaches values of close to 3.0–5.0 (postprandial range). Such acidity can hinder the use of enzyme-based sensors, since very low pHs will affect the ability of lactate dehydrogenase to catalyze the reaction.

Clearly, ss-PEC with PEDOT-type electrodes represents an advantage for LA detection, since LLA is gradually converted to pyruvate/lactate in the semiconducting electrode. Instead, in PBS, the LLA seems to be released or solubilized in the electrolyte, hindering the electrode’s ability to detect biomolecule deprotonation. On the other hand, Figure S4 shows that the linear dependence of the current density at +0.20 V in cyclic voltammograms, in which lactate oxidizes to pyruvate, is better with the square root of the scan rate than with the scan rate for PP/PEDOT-LA in both PBS and γ-PGA. This suggests that the electrochemical reaction is under the control of diffusion, independent of the electrolytic medium.

### 3.3 Viability of ss-PEC to Host and Monitor LA Conversion to Pyruvate under Electrical Stimulation

For this task, the experiments were conducted with new ss-PECs composed of PP/PEDOT-O as CE, PP/PEDOT-LA as WE, and γ-PGA hydrogel as a soft and porous semisolid electrolyte. Cyclic voltammograms were recorded at regular intervals (5, 15, 30, 60, 180, and 360 min). This study demonstrated that PP/PEDOT-O electrodes are effective carriers of LA molecules, and delivery is feasible by diffusion mechanisms and electrical pulses.

Figure 4A displays the CVs of LA reduction under diffusion processes (i.e., nonstimulated system). As can be seen, the typical oxidation peak of LA to pyruvate appeared at +0.20 V (pH ∼7.4), which started to decrease gradually from 5 to 60 min. After 180 min, the oxidation peak disappeared, suggesting the complete conversion of LA to lactate. After intercalating continuously 0.20 V positive and negative pulses of 5 ms each (15 min in total), the immediate reduction of *E*_ox_ at +0.20 V was observed (Figure 4B) in only 15 min. The adjustment of an exponentially descending curve clearly demonstrated this behavior (Figure 4C), reaching almost zero current density after 360 min. Thus, the electrical stimulus promoted a faster LA redox reaction with the highest current density sensitivity, and without voltage, the oxidation of LA to lactate was more sustained.

**Figure 4:**
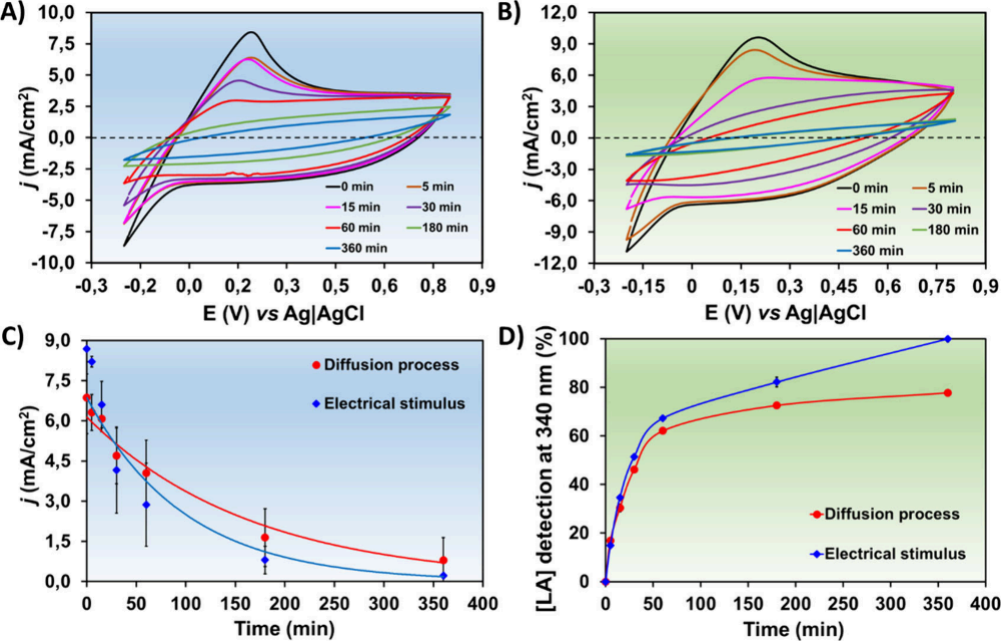
Control voltammograms of PP/PEDOT-LA (WE) in the γ-PGA hydrogel as a semisolid electrolyte in ss-PEC: A) during the LA redox reaction controlled by the diffusion process and B) stimulated by voltage pulses, with increasing time (5, 15, 30, 60, 180, and 360 min). C) Current density at +0.2 V (*E*_ox_ of LA) versus time (5, 15, 30, 60, 180, and 360 min), comparing diffusion- and voltage-induced processes. Electrical stimulus was applied using biphasic voltage pulses of ±0.2 V, with a duration of 5 ms each during 15 min total. D) NADH indirect reaction of LA from ss-PEC in Milli-Q water, detected by UV–visible absorbance at 340 nm, comparing the non-stimulated process and electrical stimulus at different times (5, 15, 30, 60, 180, and 360 min). The number of electrodes charged with LA biomolecules and tested in C) and D) was three (*n* = 3).

Finally, the successful biomolecule conversion to lactate was quantified using the indirect analytical method described in section 2.4, which is based on the spectrophotometric measurement of nicotinamide-adenine dinucleotide (NADH) formed through the catalytic action of l-lactate dehydrogenase (l-LDH) (eq 4).^14,68^ Actually, NADH does not react with LA directly; rather, it reacts with pyruvate, and lactic acid is the unique product (lactate + H^+^). Accordingly, the absorbance of NADH molecules, as measured by UV–visible spectroscopy, is directly proportional to the molar amount of the LA biomolecule that was converted to pyruvate by electrical stimulation.

4 $$\left[\mathrm{oxidation}\right]{\mathrm{CH}}_{3}\text{−}\mathrm{CO}\text{−}\mathrm{COOH}\left(\mathrm{pyruvate}\right)+\mathrm{NADH}+H^{+}\to {\mathrm{CH}}_{3}\text{−}\mathrm{CH}\left(\mathrm{OH}\right)\text{−}{\mathrm{COO}}^{-}\left(\mathrm{lactate}\right)+H^{+}$$

Figure S5 displays the calibration curve obtained measuring the absorbance at λ = 340 nm, assigned to the reaction of NADH in the presence of different concentrations of LA (from 4 to 500 mM). The calibration curve was used to quantify the total loading of LA in the PP/PEDOT-O semiconducting electrode. After 24 h of WE immersion in Milli-Q water, the average of three samples revealed a total loading of 36.9 ± 5.6 mM LA per 1 cm^2^ of the electrode carrier.

Having confirmed by different approaches that PP/PEDOT-O was successfully charged with LA biomolecules and that the bilayer electrode was able to sense the redox reaction of pyruvate in ss-PEC cells, the molecule detection was also monitored by UV–visible spectroscopy as a proof of concept for the novel ss-PEC electrochemical sensor. Figure 4D shows the two profiles of NADH detection at 340 nm, at different cell working intervals (5, 15, 30, 60, 180, and 360 min), either by diffusion or by voltage pulse-induced stimulus. The LA conversion was stabilized after 60 min of CV in the diffusion mechanism, while it followed a progressive increase in lactate conversion (indirect detection method) under an electrical stimulus, reaching 100% at 360 min. Such results evidenced once again the powerful viability of the PP/PEDOT-O semiconducting electrode and the ss-PEC cell as an effective system for LA analytical sensing.

## 4 Conclusions

In the present study, a fully organic bilayer electrode composed of flat, inert, and biocompatible PP films with a semiconducting polymer layer was strategically engineered to absorb lactic acid molecules and to convert them to a pyruvate compound, without enzymatic catalysis. The results proved that the soft electrode served as both an electro-stimulated carrier of LA and as an electrochemical sensor for the quantification of such a natural biomolecule in a semisolid electrolyte, constituted of the γ-PGA hydrogel.

The employment of a semisolid and completely organic electrochemical cell (ss-PEC), with a highly stable electrolyte (cross-linked hydrogel), proved to be more efficient in the fast release of pyruvate molecules with respect to classical three-electrode cells containing a buffer electrolyte (PBS). The hypothesis is that the permeability and acidic nature of γ-PGA are able to maintain the p*K*_a_ for the reversible reaction of LA/lactate, while liquid electrolytes, such as PBS, can only actuate as ionic transfer media.

The utilization of electrochemical stimulation offered an exponential detection of LA oxidation over increasing time, reaching 100% LA conversion to pyruvate in 6 h, proving the feasibility of the organic electrodes and the ss-PEC cell for the *in vitro* monitoring of this biomolecule. Since the determination of the lactate level is not only important in medical diagnosis to prevent some diseases but also relevant in the food and beverage industry to assess their dairy product qualities, the development of new polymeric electrodes and cells with affordable preparation and reliable LA measurements is of utmost importance to advance in those fields.

## Supplementary Material



## Notes

Additional reaction scheme of the cross-linking of the γ-PGA hydrogel with cystamine hydrochloride and complementary results (including Raman spectra, SEM micrographs, [LA] detection, and the NADH calibration curve) (PDF)
