# Supplementary material for: A Semisolid Polymer-Based Electrochemical Cell for Electrostimulated Lactate Detection
Source: ACS Omega. 2025 Dec 11;10(50):62216–26. doi: 10.1021/acsomega.5c09923 (PMC12750194; doi:10.1021/acsomega.5c09923)
Supplement: Supplementary file 1 [file ao5c09923_si_001.pdf]

A Semi-Solid Polymer-Based Electrochemical Cell for Electro-Stimulated Lactate Detection

Carolina Vitales,[1] Jordi Sans,[1,2,3] Adrián Fontana-Escartín,[1,2] Elaine Armelin,[1,2*] and Carlos Alemán[1,2,3*]

[1] *IMEM-BRT group, Departament d'Enginyeria Química, EEBE, Universitat Politècnica de Catalunya, C/ Eduard Maristany 10-14, Building I, 2nd floor, 08019, Barcelona, Spain.*

[2] *Barcelona Research Center for Multiscale Science and Engineering, Universitat Politècnica de Catalunya, C/ Eduard Maristany 10-14, Building I, basement, 08019, Barcelona, Spain.*

[3] *Institute for Bioengineering of Catalonia (IBEC), The Barcelona Institute of Science and Technology, Baldiri Reixac 10-12, 08028, Barcelona, Spain.*

Corresponding authors: elaine.armelin@upc.edu and carlos.aleman@upc.edu

2. MATERIALS and METHODS

2.5. Assembly of the semi-solid polymer-based electrochemical cell (ss-PEC)

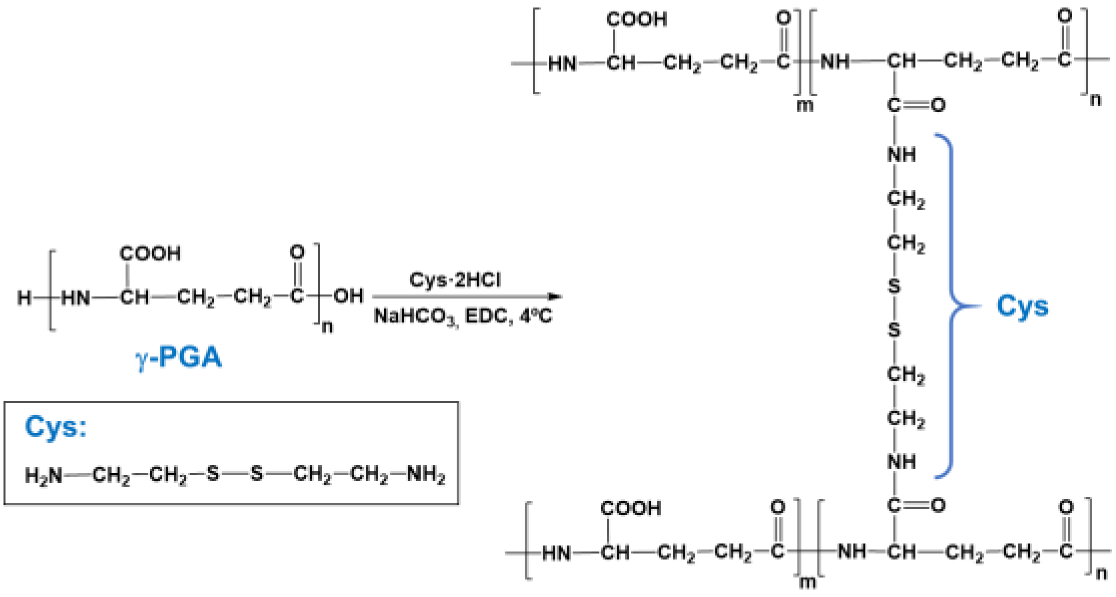

Scheme S1. Crosslinking of γ-PGA hydrogel with cystamine hydrochloride: hydrogel used as soft electrolyte in ss-PEC.

3. RESULTS and DISCUSSION

3.1. Chemical structure and morphological description of the organic conducting electrode and the polyelectrolyte employed in the construction of the ss-PEC

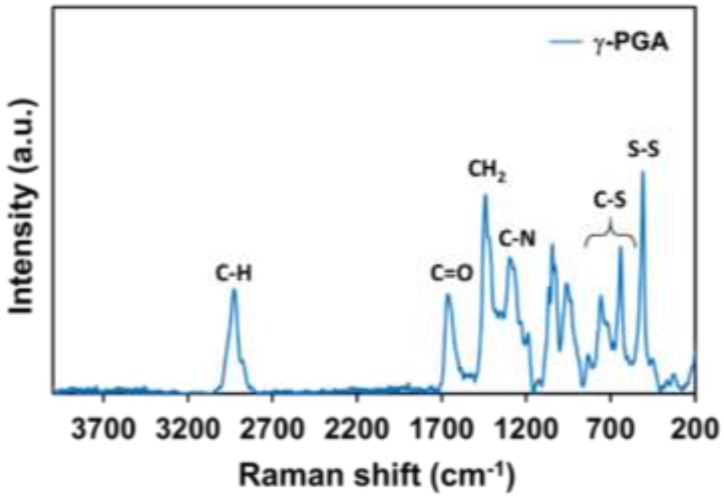

Figure S1. Raman spectrum of γ-PGA hydrogel used as semi-solid electrolyte.

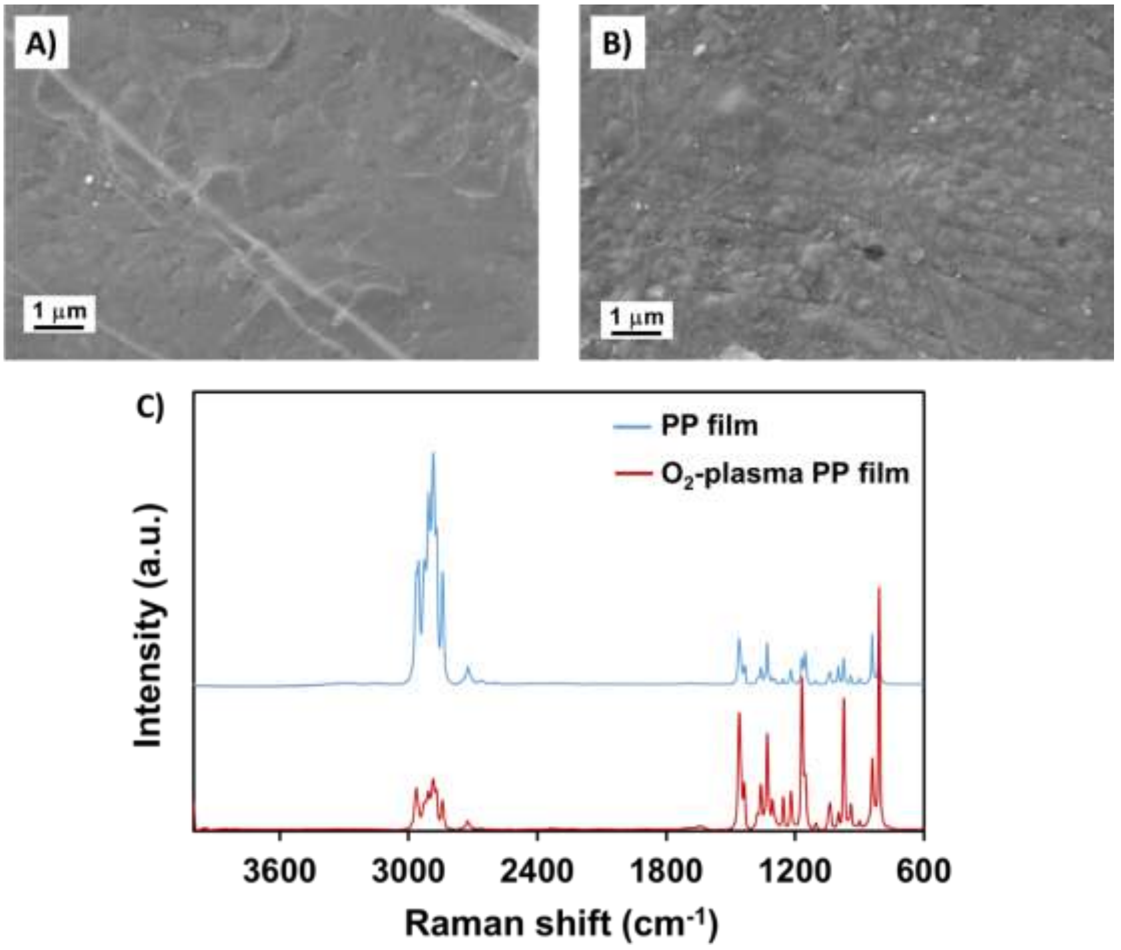

Figure S2. SEM micrographs comparison between flat PP films: A) before and B) after O_2-plasma treated surfaces, showing the visible changes on topography roughness. C) Raman spectra of PP film before and after plasma treatment.

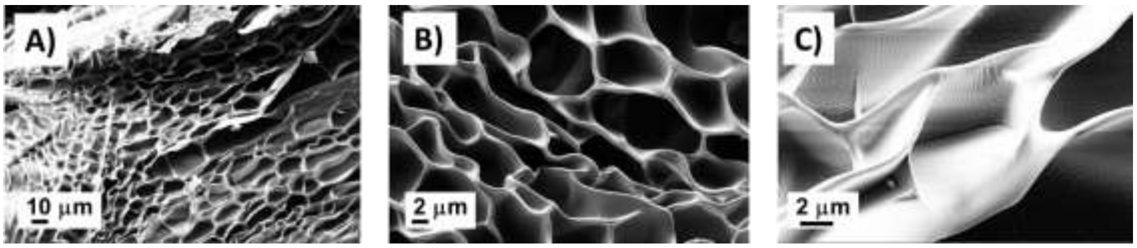

Figure S3. SEM micrographs of crosslinked γ-PGA hydrogel, used as semi-solid electrolyte for the fully organic electrochemical sensor: A) 510× magnification; B) 2500× magnification; and C) 5000× magnification.

3.2. Electrochemical activity and stability of the organic electrodes in ss-PEC

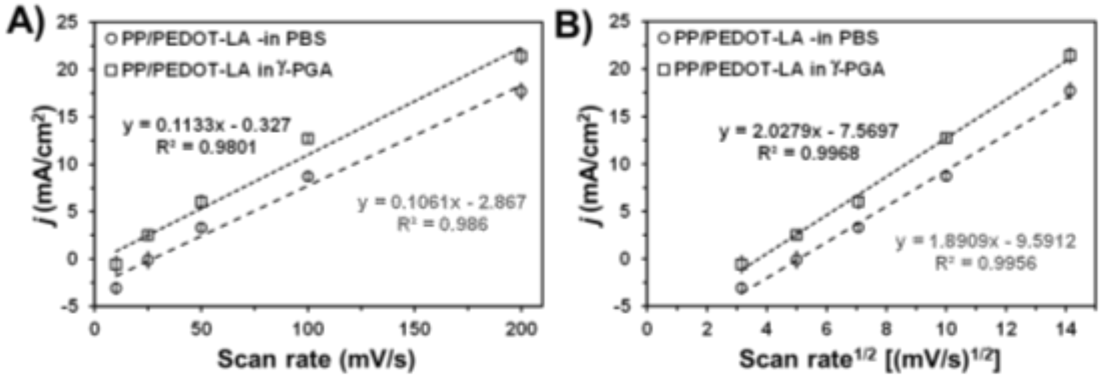

Figure S4. Variation of the current density at +0.20 V, which corresponds to the oxidation of lactate to pyruvate in cyclic voltammograms: (A) with different scan rates and (B) with the square root of the scan rates.

3.3. Viability of ss-PEC to host and to monitor the LA conversion to pyruvate under electrical stimulation

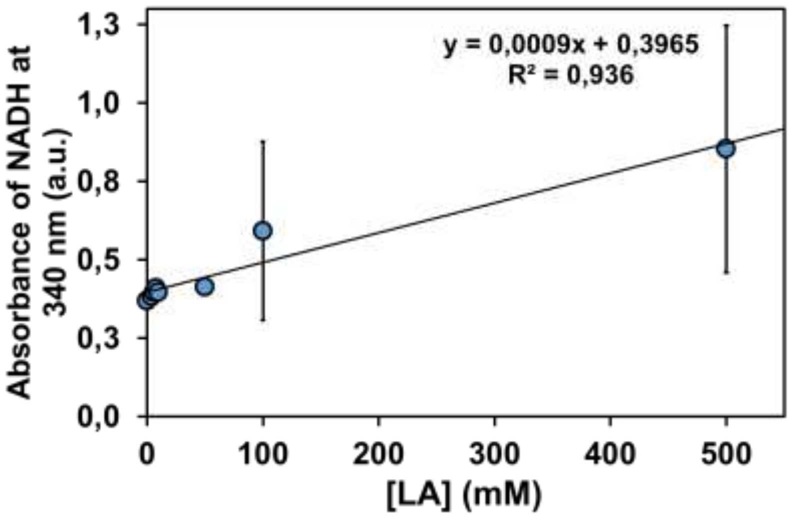

Figure S5. Calibration curve representing the absorbance of NADH at $\lambda = 340$ nm *versus* the concentration of LA reacting with NADH from the analytical kit. The LA concentration varied from 4 mM to 500 mM) ($n = 3$).
